# *CALR* mutations in a cohort of *JAK2* V617F negative patients with suspected myeloproliferative neoplasms

**DOI:** 10.1038/s41598-019-56236-x

**Published:** 2019-12-27

**Authors:** Tanja Belcic Mikic, Tadej Pajic, Matjaz Sever

**Affiliations:** 1 Department of Haematology, University Medical Centre Ljubljana, Zaloska 7, 1000 Ljubljana, Slovenia; 2 Faculty of Medicine, University of Ljubljana, Korytkova 2, 1000 Ljubljana, Slovenia; 3 Faculty of Medicine, University of Maribor, Taborska ulica 8, 2000 Maribor, Slovenia

**Keywords:** Diagnostic markers, Molecular medicine, Myeloproliferative disease

## Abstract

Suspicion of myeloproliferative neoplasms (MPNs) and especially essential thrombocythemia (ET) in primary care is often based solely on blood counts, with patients referred to a haematologist without a thorough evaluation. We retrospectively assessed the role of calreticulin gene (*CALR*) mutations in the diagnosis of MPN in this population. We studied *CALR* mutations in 524 *JAK2* V617F-negative patients with suspected MPN. Uncommon *CALR* mutations were confirmed by Sanger sequencing and searched for in the COSMIC or HGMD database. Mutations were defined as frameshift or non-frameshift mutations. *CALR* mutations were detected in 23 patients (23/524 = 4.4%). Four mutations detected in our study were newly identified mutations. Non-frameshift mutations were detected in two patients. Most patients (380/524 = 72.5%) were diagnosed with secondary conditions leading to blood count abnormalities such as iron deficiency, inflammatory and infectious diseases, malignancy and hyposplenism. Nine patients (9/23 = 39%) were retrospectively diagnosed with ET based on *CALR* mutation confirmation. Two patients with non-frameshift *CALR* mutations were diagnosed with reactive thrombocytosis and MPN unclassifiable, respectively. Our study showed that *CALR* mutations are important, non-invasive diagnostic indicators of ET and can aid in its diagnosis. Moreover, the type of *CALR* mutation must be accurately defined, as non-frameshift mutations may not be associated with ET. Finally, *CALR* mutation detection should be reserved for patients with high suspicion of clonal haematological disease.

## Introduction

In 2013, mutations in the calreticulin gene (*CALR*) were identified in two Philadelphia chromosome (Ph)-negative myeloproliferative neoplasms (MPNs), essential thrombocythemia (ET) and primary myelofibrosis (PMF)^1,2^. In addition, *CALR* mutations have been detected rarely in chronic myelomonocytic leukaemia (CMML)^1^, myelodysplastic syndrome/myeloproliferative neoplasm (MDS/MPN)^3^, and a few myelodysplastic syndrome (MDS) patients, mainly with refractory anaemia with ring sideroblasts, refractory anaemia, and refractory anaemia with excess blasts^4^. The presence of a *CALR* mutation has been described as an exceptional finding in cases of polycythaemia vera (PV) with an unknown pathogenic role^5^. Since its discovery, more than 50 mutations in the *CALR* gene have been detected as occurring in exon 9, inducing a + 1 (−1 + 2) frameshift. Only mutations leading to this +1 frameshift are considered to be pathogenic. Other mutations are usually germline variants of *CALR* and are not known to be pathogenic^6^.

Calreticulin (CALR) is a chaperone protein involved in many cellular processes in the cytoplasm and in the endoplasmic reticulum (ER). In the ER, it acts as a calcium binding protein^7^. It has three domains with oncogenic properties reflected by the C terminal domain^8^. The C terminal domain of mutant CALR is devoid of the KDEL motif, which is important for protein retention in the ER. The mutated C terminal domain contains a new amino acid sequence that bears positive charges^2^. The exact mechanism underlying the MPN phenotype in patients with mutant CALR remains unclear. Most important is the specific activation of the thrombopoietin receptor (TpoR/MPL) and uncontrolled activation of the JAK2/STAT pathway in the MAP kinase pathway via TpoR^9^.

The two most frequent *CALR* mutations are a 52 bp deletion (p.L367fs*46), also called type 1, and a 5 bp insertion (p.K385fs*47), also called type 2. According to these structural changes, the other mutations have been classified as type 1–like or type 2–like using algorithms based on the preservation of an α helix close to wild type CALR^10^.

The diagnostic value of *CALR* mutation confirmation has been defined only recently by including *CALR* mutations in the diagnostic criteria for ET/PMF in the 2016 revision to the World Health Organization (WHO) classification of myeloid neoplasms and acute leukaemia^11^.

Suspicion for MPN in primary care is often based solely on complete blood cell count, with patients referred to a haematologist without a thorough history, examination or laboratory testing.

The aim of our study was to retrospectively confirm the diagnostic importance of *CALR* mutations in *JAK2* V617F-negative patients with suspected MPN based on clinical and/or laboratory parameters who were referred to the Department of Haematology by general practitioners. Furthermore, our goal was to determine possible novel *CALR* mutations and their clinical effects, including thrombo-haemorrhagic complications in *CALR*-positive patients.

## Patients and Methods

### Study design

*CALR* mutations were studied in *JAK2* V617F*-*negative patients with suspected MPN based on clinical and/or laboratory parameters (listed in Table 1) who were referred by general practitioners and then examined at the Department of Haematology, University Medical Centre Ljubljana, between April 7, 2011 and September 13, 2016 (inclusion and exclusion criteria are listed in Table 2). The study was retrospective and nonrandomized and was approved by the National Medical Ethics Committee, Ministry of Health, Republic of Slovenia, on November 14, 2017. All experiments were performed in accordance with relevant guidelines and regulations, and informed consent was obtained from all the participants.

**Table 1:** Clinical and/or laboratory parameters leading to suspicion of MPN (at least one parameter was mandatory for suspicion) – suspicion was defined by general practitioners

1. Palpable splenomegaly
2. Unexplained arterial/venous thrombosis
3. Thrombocytosis (Plt* count ≥ 410 × 10^9^/L)
4. Erythrocytosis (Hb** ≥ 150 g/l in females and ≥170 g/l in males)
5. Leucocytosis (WBC^+^ count ≥ 10 × 10^9^/L)
6. Anaemia (Hb below 100 g/l)

*Platelet, **Haemoglobin, ^+^White blood cell.

**Table 2:** Study inclusion and exclusion criteria.

**Inclusion criteria**
Age ≥ 18 years
Date of first examination at the Department of Haematology between April 7, 2011 and September 13, 2016
Clinical and/or laboratory suspicion of MPN
Agreement to undergo genetic testing
**Exclusion criteria**
*JAK*2 V617F mutation confirmed by genetic testing
Inability to obtain an appropriate DNA sample from the available material at the blood sample library

We collected history, clinical and laboratory data from our institutional database. The following data were collected from our institutional database: age at the date of first examination, gender, haemoglobin level, leukocyte blood count, and platelet count, the presence of splenomegaly or unexplained arterial/venous thrombosis and haematological diagnosis defined at the Department of Haematology. *CALR*-positive patients were re-diagnosed after *CALR* mutation confirmation based on available clinical and laboratory data. ET was diagnosed by the 2008 WHO criteria^12^. In patients without bone marrow samples, modified WHO criteria were used^13^. Other MPN subtypes were diagnosed according to the 2008 WHO criteria^12^. *CALR*-positive patients were screened for thrombo-haemorrhagic complications according to the available data from follow-up visits at the Department of Haematology.

### Molecular-genetic testing

*CALR* mutations were analysed in patients’ neutrophil granulocytes. Granulocytes were isolated from venous blood samples by Ficoll density gradient centrifugation followed by a red blood cell lysis procedure^14^. DNA was isolated from granulocytes by a QIAamp DNA Mini Kit from Qiagen (USA).

The detection of *CALR* exon 9 mutations was performed with fluorescence-based quantitative real-time PCR (qPCR) and post-qPCR analysis with the High Resolution Melting-Curve Analysis (HRM) method. A MeltDoctor HRM MasterMix (Applied Biosystems, Thermo Fischer Scientific, USA) and primer sets published previously^2^ were used for the qPCR and HRM analysis. Both were performed according to the manufacturer’s instructions on an ABI PRISM ViiA 7 Real-Time PCR system (Applied Biosystems, Thermo Fisher Scientific). Specifically, samples of 20 ng DNA were amplified by qPCR using the following thermal cycling protocol: 95 °C, 10 minutes for 1 cycle, followed by 50 cycles of 95 °C for 10 seconds and 62.5 °C for 60 seconds. Melt curve/dissociation stage was carried out immediately after qPCR according to the manufacturer’s instructions (Applied Biosystems, Thermo Fischer Scientific). We included two positive controls (NM_004343.3 (CALR): c.1099_1150del52, p.(Leu367Thrfs*46) (52 bp deletion or type 1 mutation) and NM_004343.3 (CALR): c.1154_1155insTTGTC, p.(Lys385Asnfs*47) (5 bp insertion or type 2 mutation)), as well as wild-type and non-template controls, in the qPCR and HRM setup. qPCR products were visualized on a 4% agarose E-gel by the E-Gel Precast Agarose Electrophoresis System (Invitrogen, Thermo Fischer Scientific, USA). The most frequent mutations, the 52 bp deletion (type 1 mutation) and the 5 bp insertion (type 2 mutation), were confirmed by a typical HRM melt curve and band pattern on the E-gel. An unusual HRM melt curve and/or band pattern on the E-gel was indicative of a different genetic variant, which was confirmed by Sanger sequencing according to the recommended protocol from Applied Biosystems, Thermo Fischer Scientific Company. Mutations were defined by type as type 1, type 2, type 1-like or type 2-like by the AGADIR-derived predicted helix propensity scale (AGADIR score)^15–17^.

The Catalogue of Somatic Mutations in Cancer (COSMIC)^18^ and The Human Gene Mutation Database (HGMD)^19^ were used to determine the impact of new genetic variants of the *CALR* gene.

### Statistical analysis

Numerical variables are summarized by their median and range and categorical variables by count and relative frequency (%). Statistical analysis was performed using the JASP 0.9.2.0 statistical program.

## Results

### Patient demographics

A total of 524 patients were screened for the presence of *CALR* mutations. Of these patients, 292 were female and 232 were male. The median age at diagnosis was 55 years. Demographic and laboratory parameters of patients suspected of MPN are presented in Table 3.

**Table 3:** Demographic and laboratory parameters of patients suspected of MPN.

Total number of analysed patients	524
Sex (male/female)	232/292
Age at onset, years, median (range)	55 (18–90)
Hb* above 165 g/l in females, *n* (%)	21 (7.19)
Hb* above 185 g/l in males, *n* (%)	15 (6.46)
WBC** count above 10.0 × 10^9^/l, *n* (%)	180 (34.3)
Plt^+^ count above 450 × 10^9^/l, *n* (%)	94 (17.9)
Palpable splenomegaly, *n* (%)	13 (2.48)
Unexplained arterial/venous thrombosis, *n* (%)	3 (0.57)

*Haemoglobin, **White blood cell, ^+^Platelet.

At the Department of Haematology, most patients (380/524 = 72.5%) were identified with secondary, non-clonal conditions, resulting in erythrocytosis, leucocytosis or thrombocytosis. The most common secondary conditions or factors were iron deficiency, infectious and inflammatory diseases, hyposplenism, malignancy, smoking, recent surgery, use of corticosteroids and chronic hypoxia. Diagnosis was defined after the examination and diagnostic workup at the Department of Haematology. Twenty-six patients (26/524 = 4.96%) were diagnosed with different types of MPN according to the 2008 WHO criteria^12^. Two patients (2/524 = 0.38%) were diagnosed with chronic myelomonocytic leukaemia (CMML). Thirty-two patients (32/524 = 6.1%) were suspected of having clonal ET but were not diagnosed at the time of examination. The list of diagnoses in MPN-suspected patients is presented in Supplementary Table S1.

### *CALR* mutation detection

*CALR* mutations were detected in 23 patients (23/524 = 4.4%). The types of *CALR* mutations found in our cohort of patients are listed in Table 4. Four mutations that were detected in our study were newly identified mutations that had not yet been published in the COSMIC or HGMD database. They are marked in bold in Table 4. Two *CALR* mutations detected in our study are non-frameshift mutations and are underlined in Table 4.

**Table 4:** Types of *CALR* mutations in our patients. All mutations were searched for in the COSMIC or the HGMD database and are labelled with their COSMIC or HGMD identity number if it existed at the time of search. All mutations that were not found in the COSMIC and HGMD databases do not have a COSMIC or HGMD identity number and are, to our knowledge, newly identified mutations that have not yet been defined. Newly identified mutations in our patients are marked in bold. Non-frameshift mutations are underlined.

Patient number	*CALR* genetic variant	Type of mutation	COSMIC or HGMD ID	Confirmed somatic (database COSMIC)	Diagnosis at the Department of Haematology
1	NM_004343.3 (CALR):c.1127_1145del19, p.(Arg376Glnfs*48)	Type 2-like	None	No, status unknown	ET 2008 WHO
2	NM_004343.3 (CALR):c.1099_1150del52, p.(Leu367Thrfs*46)	Type 1	COSM1738055	Yes	High suspicion for ET
3	NM_004343.3 (CALR):c.1099_1150del52, p.(Leu367Thrfs*46)	Type 1	COSM1738055	Yes	ET 2008 WHO
4	NM_004343.3 (CALR):c.1099_1150del52, p.(Leu367Thrfs*46)	Type 1	COSM1738055	Yes	PMF 2008 WHO
5	NM_004343.3 (CALR):c.1154_1154delAinsGTTGTC, p.(Lys385Serfs*47)	Type 2-like	None	No, status unknown	ET 2008 WHO
6	NM_004343.3 (CALR):c.1154_1155insTTGTC, p.(Lys385Asnfs*47)	Type 2	COSM1738056	Yes	High suspicion for ET
7	NM_004343.3 (CALR):c.1099_1150del52, p.(Leu367Thrfs*46)	Type 1	COSM1738055	Yes	ET 2008 WHO
8	NM_004343.3 (CALR):c.1099_1150del52, p.(Leu367Thrfs*46)	Type 1	COSM1738055	Yes	PMF 2008 WHO
9	NM_004343.3 (CALR):c.1154_1154delAinsTTTATC, p.(Lys385Ilefs*47)	Type 2-like	None	No, status unknown	High suspicion for ET
10	NM_004343.3 (CALR):c.1099_1150del52, p.(Leu367Thrfs*46)	Type 1	COSM1738055	Yes	High suspicion for ET
11	NM_004343.3 (CALR):c.1099_1150del52, p.(Leu367Thrfs*46)	Type 1	COSM1738055	Yes	High suspicion for ET
12	NM_004343.3 (CALR):c.1092_1143del52, p.(Leu367Thrfs*46)	Type 1	COSM1738055	Yes	High suspicion for ET
13	NM_004343.3 (CALR):c.1092_1143del52, p.(Leu367Thrfs*46)	Type 1	COSM1738055	Yes	ET 2008 WHO
14	NM_004343.3 (CALR):c.1092_1143del52, p.(Leu367Thrfs*46)	Type 1	COSM1738055	Yes	PMF 2008 WHO
15	NM_004343.3 (CALR):c.1154_1155insTTGTC, p.(Lys385Asnfs*47)	Type 2	COSM1738056	Yes	ET 2008 WHO
16	NM_004343.3 (CALR):c.1092_1143del52, p.(Leu367Thrfs*46)	Type 1	COSM1738055	Yes	PMF 2008 WHO
17	NM_004343.3 (CALR):c.1092_1143del52, p.(Leu367Thrfs*46)	Type 1	COSM1738055	Yes	ET 2008 WHO
18	NM_004343.3 (CALR):c.1142_1144delAGG, p.(Glu381del)	In-frame mutation	CD176098	Status unknown, probably germline	MPN-unclassifiable (MPN-U)
19	NM_004343.3 (CALR):c.1194 G > T, p.(Glu398Asp)	Missense mutation	COSM1738023	No, status unknown	Reactive thrombocytosis
20	NM_004343.3 (CALR):c.1132_1153del22, p.(Glu378Argfs*45)	Type 2-like	None	No, status unknown	High suspicion for ET
21	NM_004343.3 (CALR):c.1114_1144del31, p.(Glu372Glnfs*48)	Type 2-like	COSM3734991	No, status unknown	High suspicion for ET
22	NM_004343.3 (CALR):c.1092_1143del52, p.(Leu367Thrfs*46)	Type 1	COSM1738055	Yes	High suspicion for ET
23	NM_004343.3 (CALR):c.1099_1150del52, p.(Leu367Thrfs*46)	Type 1	COSM1738055	Yes	CMML

Sixteen mutations (16/23 = 69.6%) that were found in the COSMIC database were confirmed somatic. All other mutations were of unidentified status.

The most common type of *CALR* mutation was a 52 bp deletion (type 1 mutation) that was diagnosed in fourteen patients (14/23 = 60.9%). A type 2 mutation (5 bp insertion) was present in two patients (2/23 = 8.7%). Other *CALR* mutations were present in 7 patients (7/23 = 30.4%) and were sub-classified by the AGADIR‐derived predicted helix propensity score as described in the literature^17^. The AGADIR score was 17.50 for type 1, 40.02 for type 2 and 33.62 for wild-type CALR. CALR variants with an AGADIR scale of 26% or less were classified as type 1-like and CALR variants with an AGADIR scale of 30% or more as type 2-like. According to the AGADIR scale, five patients were diagnosed with type 2-like mutations (Table 5). Two patients did not have a frameshift mutation and were not sub-classified as type 1- or type 2-like.

**Table 5:** Frameshift CALR variant classification using the alpha helix propensity model (AGADIR score) in patients with non-type 1/2 mutant CALR variants. Subsequently new amino acids are shown in bold, common 3′ end are shown in italics.

No. of patient (*n* = 5)	CALR variant	AGADIR helix propensity score (T = 25 °C)	Nucleotide Change	Amino Acid Sequence	Amino Acid Change
1	Type 2-like	32.05	c.1127_1145del19	p.(Arg376Glnfs*48)	AAEKQMKDKQDEEQRLKEEEEDK K**QRTRRMMRTKM***RMRRMRRTRRKMRRKMSPARPRTSCREACLQGWTEA*
2	Type 2-like	37.24	c.1154_1154delAinsGTTGTC	p.(Arg376Glnfs*48)	AAEKQMKDKQDEEQRLKEEEEDKKRKEEEEAED**SCRRMMRTKM***RMRRMRRTRRKMRRKMSPARPRTSCREACLQGWTEA*
3	Type 2-like	46.58	c.1154_1154delAinsTTTATC	p.(Lys385Ilefs*47)	AAEKQMKDKQDEEQRLKEEEEDKKRKEEEEAED**IYRRMMRTKM***RMRRMRRTRRKMRRKMSPARPRTSCREACLQGWTEA*
4	Type 2-like	31.05	c.1132_1153del22	p.(Glu378Argfs*45)	AAEKQMKDKQDEEQRLKEEEEDKKRK**RRMMRTKM***RMRRMRRTRRKMRRKMSPARPRTSCREACLQGWTEA*
5	Type 2-like	32.43	c.1114_1144del31	p.(Glu372Glnfs*48)	AAEKQMKDKQDEEQRLKEEE**QRTRRMMRTKM***RMRRMRRTRRKMRRKMSPARPRTSCREACLQGWTEA*
	Type 1	17.50	c.1092_1143del52	p.(Leu367Thrfs*46)	AAEKQMKDKQDEEQR**TRRMMRTKM***RMRRMRRTRRKMRRKMSPARPRTSCREACLQGWTEA*
	Type 2	40.02	c.1154_1155insTTGTC	p.(Lys385Asnfs*47)	AAEKQMKDKQDEEQRLKEEEEDKKRKEEEEAED**NCRRMMRTKM***RMRRMRRTRRKMRRKMSPARPRTSCREACLQGWTEA*
	Wild-type	33.62	Wild-type	Wild-type	AAEKQMKDKQDEEQRLKEEEEDKKRKEEEEAEDKEDDEDKDEDEEDEEDKEEDEEEDVPGQAKDEL

### *CALR*-positive patients

The most common diagnosis in *CALR*-positive patients was ET, which was present in 16 patients (16/23 = 69.6%). Nine patients (9/23 = 39%) were retrospectively diagnosed with ET according to modified WHO ET criteria based on clinical and laboratory findings and *CALR* mutation confirmation. In other patients, the *CALR* mutation did not affect haematological diagnosis. The list of haematological diagnoses in the *CALR*-positive patients is presented in Table 6.

**Table 6:** Haematological diagnoses in CALR-positive patients.

Diagnosis	No. of patients
ET by 2008 WHO criteria^12^	7
ET by modified WHO criteria^13^	9
PMF	4
CMML	1
MPN- U	1
Reactive thrombocytosis	1

Seven *CALR*-positive patients (7/23 = 30%) developed thrombo-haemorrhagic complications. Five patients (5/23 = 21.7%) developed thrombotic complications, and two patients (2/23 = 8.7%) developed haemorrhagic complications. Thrombo-haemorrhagic complications occurred mostly in patients diagnosed with ET (6/7 = 85.7%), and one complication occurred in a patient diagnosed with PMF. Patients with thrombo-haemorrhagic complications were mostly diagnosed with type 1 mutation (5/7 = 71.4%), and two patients were diagnosed with type 2 and type 2-like mutations. The median follow-up time was 693 days (range: 196–1270 days). A list of complications, haematological diagnoses and the times of complication is presented in Table 7.

**Table 7:** Thrombo-haemorrhagic complications in *CALR*-positive patients, their haematological diagnoses and the times of complication.

No. of patient (n = 7)	Type of complication	Time of complication and antithrombotic treatment	Haematological diagnosis
*1*	DVT*	After the diagnosis, on treatment with aspirin	ET 2008 WHO
*2*	Subconjunctival bleeding	After the diagnosis, on treatment with aspirin	ET 2008 WHO
3	Vitreous bleeding	After the diagnosis, on treatment with warfarin	PMF 2008 WHO
4	Ischaemic stroke	Before the diagnosis	ET modified WHO
5	Ischaemic stroke	Before the diagnosis	ET modified WHO
6	DVT*	Before the diagnosis	ET 2008 WHO
7	Thrombophlebitis	Before the diagnosis	ET 2008 WHO

*Deep venous thrombosis.

## Discussion

In our study, we retrospectively detected the presence of *CALR* mutations in a cohort of 524 *JAK2* V617F-negative patients who presented with clinical and/or laboratory suspicion of MPN with the main goal of determining the diagnostic value of this detection. During the observed period, *CALR* mutations had not been routinely investigated in patients suspected of MPN in our centre, as they have only recently been discovered^1,2^. In contrast, the *JAK2* V617F mutation has already been analysed routinely since its discovery in 2005^20^. Therefore, *JAK2* V617F-positive patients were excluded from the study. An *MPL* mutation is a relatively rare finding in MPN patients and is mostly present in patients with either ET or PMF^21^. In our laboratory, *MPL* testing was included in the diagnostic algorithm in patients with suspected MPN only recently and is carried out only after excluding *JAK2* V617F and *CALR* mutations. Therefore, *MPL* status was not defined in all our patients at the time of the first examination.

Our centre is a university medical hospital serving an area with approximately 1.000.000 inhabitants and is a referral centre for haematological malignancies. All patients referred to the haematology department were first evaluated by general practitioners without a detailed knowledge in the field of haematological malignancies. As presented in Table 3 only a proportion of patients referred to the Department of Haematology had pathological clinical and/or laboratory parameters suspicious of MPN at the time of first examination. Therefore, most patients who were referred to the Department of Haematology were individuals without a haematological malignancy, as we determined that 380 patients (380/524 = 72.5%) were MPN free. These patients were diagnosed with secondary changes in the peripheral blood content caused by different conditions, such as iron deficiency, infectious and inflammatory diseases, hyposplenism, malignancy, smoking, recent surgery, use of corticosteroids and chronic hypoxia. Accordingly, the number of *CALR*-positive patients in our cohort was low. We identified 4.4% of patients suspected for MPN as *CALR* positive, most of whom were diagnosed with either ET or PMF. Most similar studies analysed the number of *CALR*-positive patients with confirmed MPN; therefore, the number of *CALR*-positive patients was much higher, ranging from 12 to more than 20%^22–26^. Based on our data, it would be beneficial to improve the diagnostic approach to patients with increased levels of one or more blood cell lineages, as in most cases, this is not caused by haematological malignancy. Patients should be routinely screened for the most common causes of secondary erythrocytosis, thrombocytosis, and leucocytosis, and blood count should be repeated at least once at follow-up visits before referral to the Department of Haematology. Molecular genetic testing should be reserved for patients with high suspicion of clonal haematological disease and not performed in all patients referred to the Department of Haematology.

*CALR* mutations are commonly identified in *JAK2* V617F-negative patients with ET^1,2^. In our centre, only a minority of *CALR*-positive patients (7/23 = 30%) were diagnosed with ET according to the 2008 WHO criteria at the time of the first examination. The main reason for this was the relative reservation towards bone marrow examination in patients with moderate thrombocytosis and a low risk of thrombotic complications. However, this means we could have underestimated the number of patients with clonal thrombocytosis. By confirming the presence of *CALR* mutations, we were able to retrospectively diagnose 9 patients with ET according to the modified WHO criteria for ET^13^. Although all of these patients had follow-up at our department due to high suspicion of clonal thrombocytosis, it is of great prognostic and therapeutic benefit to be able to confirm the diagnosis of ET by a non-invasive procedure such as molecular-genetic testing. *CALR* mutations are therefore important diagnostic hallmarks for ET as has also been confirmed recently in the literature^27,28^.

All patients with PMF were diagnosed according to the WHO criteria at the time of the examination at the Department of Haematology. *CALR* mutation identification retrospectively confirmed the diagnosis but had no direct diagnostic or therapeutic impact.

CMML is a subtype of MDSs/MPNs and not strictly MPNs^12^. *CALR* mutations in patients with CMML are extremely rare and do not play an important role in its pathogenesis. A group study by Zamora *et al*. showed that only 1 out of 174 patients with CMML presented with a *CALR* mutation^29^. The patient in our study who was diagnosed with CMML type one and later progressed to myelofibrosis might have been misdiagnosed at the time of the first examination, as PMF and CMML share many common features, including monocytosis and bone marrow fibrosis. In a recent study by Hu *et al*., a more accurate analysis enabled many patients diagnosed with CMML to be reclassified as having PMF, showing that a more in-depth analysis should be performed to make an accurate diagnosis, especially in patients with molecular biomarkers typical for ET/PMF^11,30^.

More than 50 frameshift *CALR* mutations have been described, and all are located in exon 9 of the *CALR* gene. There are two main types of *CALR* mutations: type 1 (a 52-bp deletion; p.L367fs*46) and type 2 (a 5-bp TTGTC insertion; p.K385fs*47). Based on their molecular characteristics, other mutations can be grouped as type 1- like and type 2- like^31^. In our study, the most common type of *CALR* mutation was type 1, which is consistent with the data from the literature^2,16,32^. Mutations that are neither type 1 nor type 2 should be sub-classified as type 1-like or type 2-like, as this may have an impact on clinical phenotype and, in the case of PMF, even survival^17^. Type 1-like and type 2-like mutations in our study were defined by using AGADIR^15^, a statistical approximation algorithm that calculates helix propensity for the 31 unique amino acid sequences that are altered by *CALR* mutations^16^. This algorithm has been used in similar studies and represents an important tool in *CALR* mutation sub-classification^16,17^. It seems extremely important to properly define the type of *CALR* mutation, as this also has a diagnostic impact. It is known that only mutations leading to a + 1 (−1 + 2) frameshift of the reading frame are pathogenic^6^. Other *CALR* mutations can be germline variants of *CALR* with an unknown clinical significance. In our study, non-frameshift *CALR* mutations were detected in two patients. One of these patients was diagnosed with reactive thrombocytosis at the time of examination at the Department of Haematology and was retrospectively defined as *CALR-*positive. A detailed analysis confirmed a missense *CALR* mutation that results in the substitution of glutamic acid with aspartic acid at amino acid position 398. The same mutation was discovered in a patient with WHO-defined chronic neutrophilic leukaemia (CNL) in a study by Lasho *et al*. His study concluded that *CALR* mutations that do not result in a generation of a distinct C-terminus are suggestive of a different pathogenic mechanism that might be yet unknown^33^. In our study, we assumed that our patient did not develop a *CALR* mutation that would imply a clonal haematological disease. According to the patient’s history, clinical examination and laboratory findings, it was mostly suggestive of reactive thrombocytosis. At the time of writing this article, the patient was in good health without any possible complications associated with clonal haematological disease.

Another patient with a non-frameshift *CALR* mutation was diagnosed with MPN-U. This patient carried a germline in-frame deletion in the *CALR* gene (NM_004343.3 (CALR):c.1142_1144delAGG; p.(Glu381del)), which had already been recognized in a symptomatic patient with MPN^34^. Although the KDEL motif was preserved, the deletion of one amino acid (p.(Glu381del)) led to the alteration of the secondary structure of the protein as well as the three-dimensional structure, which led to the conclusion of the pathogenic nature of this in-frame deletion^23,34,35^. Our patient with MPN-U was unfortunately lost to follow-up, and an exact haematological diagnosis or possible complications could not be defined. However, laboratory findings were suggestive of a clonal haematological disease.

In our study, we discovered four novel *CALR* mutations in exon 9 that, to our knowledge, have not yet been registered in the COSMIC (https://cancer.sanger.ac.uk/cosmic) or HGMD (http://www.hgmd.cf.ac.uk) database. The COSMIC database is the world’s largest expert-curated database of somatic mutations in human cancer. It describes over 4 million coding mutations^36^. The HGMD database represents an attempt to collate all known (published) gene lesions responsible for human inherited diseases^19^. The novel mutations that were defined in our study are NM_004343.3 (CALR):c.1127_1145del19, p.(Arg376Glnfs*48), NM_004343.3 (CALR):c.1154_1154delAinsGTTGTC, p.(Lys385Serfs*47), NM_004343.3 (CALR):c.1154_1154delAinsTTTATC, p.(Lys385Ilefs*47), and NM_004343.3 (CALR):c.1132_1153del22, p.(Glu378Argfs*45). All these mutations were type 2-like mutations.

ET and PMF are associated with an increased risk of thrombotic and thromboembolic events, which represent important causes of morbidity and mortality^37^. Reducing the risk of thrombotic and thromboembolic complications is one of the most important goals of treatment, especially in patients with ET^38^. These patients are also at higher risk of bleeding complications, which may be related to complications of treatment or acquired von Willebrand syndrome (AVWS) due to extreme thrombocytosis (platelets > 1000 × 10^9^/L)^28,39,40^. The risk of thrombosis in patients with ET exceeds 20%^41^. In a Swedish study, 35% of patients with ET developed vascular complications^42^. In PMF, thrombotic events are about as common as in ET^43^. The prevalence for thrombotic complications in patients with PMF ranges from 7 to 30%^44–46^. In our study, the prevalence of thrombotic complications in *CALR*-positive patients was 30%. However, three patients had developed a thrombotic complication more than 10 years before MPN diagnosis was suspected. The true prevalence of thrombotic complications in our study was therefore lower. One patient developed vitreous haemorrhage which was a result of inadequate anticoagulation therapy with warfarin. Neither of the two patients with a bleeding complication had extreme thrombocytosis at the time of complication. Compared to *JAK2* V617F and *MPL* mutations, *CALR* is a favourable mutation and is associated with a lower incidence of thrombotic events^1,2,47^. Most *CALR*-positive patients who developed thrombo-haemorrhagic complications were diagnosed with type 1 mutations (71.4%). As already shown, patients with type 1-like mutations had a higher risk of thrombosis compared to patients with type 2-like mutations^10^.

*CALR* mutations are currently known to be one of the three major mutation types, in addition to *JAK2* V617F and the *MPL* mutation, in patients with ET or PMF. However, there were still 10–15% of patients with ET or PMF with an unknown molecular genetic marker underlying the disease. These patients are termed ‘triple negative’^48^. In these patients, novel molecular biomarkers have been searched by sequencing coding exons in myeloid cancer genes, which has shown promising results. This may provide a personalized approach to diagnosis in patients with MPN^49^.

*CALR* mutations in MPN patients are also under investigation for their therapeutic potential^50–52^. *CALR* exon 9 mutations could be targets for cancer immune therapy, as they have been shown to act as immunogenic neo-antigens^51^. In the treatment of MPN, it could be beneficial to combine *CALR* vaccines with immunomodulatory treatments^53^ such as interferon-alpha (IFN-α)^54^ or programmed death 1 ligand (PD-L1)^55^ as a combinatorial cancer vaccination^53^. *CALR* mutations, in addition to being important diagnostic and prognostic markers in patients with MPN, could become an important therapeutic target in a subgroup of patients with MPN in the future.

## Conclusion

As a non-invasive test, detection of *CALR* mutations is important in the diagnosis of ET, especially in cases where bone marrow examination is not available or unwarranted. The type of *CALR* mutation must be accurately defined, as some *CALR* mutations may not be associated with ET. *CALR* mutation detection should be reserved for patients with high suspicion of clonal haematological disease. In the future, *CALR* exon 9 mutations, as immunogenic neo-antigens, could be targets for cancer immune therapy.

## Supplementary information

Supplementary Information 

## Data Availability

The datasets generated and analysed during the current study are available from the corresponding author on reasonable request.

## References

[1] Nangalia J (2013). Somatic CALR mutations in myeloproliferative neoplasms with nonmutated JAK2. N Engl J Med.

[2] Klampfl T (2013). Somatic mutations of calreticulin in myeloproliferative neoplasms. N Engl J Med.

[3] Heuser M (2014). Low frequency of calreticulin mutations in MDS patients. Leukemia.

[4] Hou HA, Kuo YY, Chou WC, Chen PH, Tien HF (2014). Calreticulin mutation was rarely detected in patients with myelodysplastic syndrome. Leukemia.

[5] Broseus J, Park JH, Carillo S, Hermouet S, Girodon F (2014). Presence of calreticulin mutations in JAK2-negative polycythemia vera. Blood.

[6] Vainchenker W, Kralovics R (2017). Genetic basis and molecular pathophysiology of classical myeloproliferative neoplasms. Blood.

[7] Raghavan M, Wijeyesakere SJ, Peters LR, Del Cid N (2013). Calreticulin in the immune system: ins and outs. Trends in immunology.

[8] Marty C (2016). Calreticulin mutants in mice induce an MPL-dependent thrombocytosis with frequent progression to myelofibrosis. Blood.

[9] Chachoua I (2016). Thrombopoietin receptor activation by myeloproliferative neoplasm associated calreticulin mutants. Blood.

[10] Pietra D (2016). Differential clinical effects of different mutation subtypes in CALR-mutant myeloproliferative neoplasms. Leukemia.

[11] Arber DA (2016). The 2016 revision to the World Health Organization classification of myeloid neoplasms and acute leukemia. Blood.

[12] Vardiman JW (2009). The 2008 revision of the World Health Organization (WHO) classification of myeloid neoplasms and acute leukemia: rationale and important changes. Blood.

[13] Harrison CN (2010). Guideline for investigation and management of adults and children presenting with a thrombocytosis. British journal of haematology.

[14] Pajič, T., Kovačič, L., Mlakar, U. The thrombopoietin receptor W515L and W515K mutations detection in patients with essential thrombocythemia. *Zdrav Vestn* **81** (2012).

[15] Munoz, V. & Serrano, L. Development of the multiple sequence approximation within the AGADIR model of alpha-helix formation: comparison with Zimm-Bragg and Lifson-Roig formalisms. *Biopolymers* **41**, 495–509, 10.1002/(sici)1097-0282(19970415)41:5<495::Aid-bip2>3.0.Co;2-h (1997). 10.1002/(SICI)1097-0282(19970415)41:5<495::AID-BIP2>3.0.CO;2-H 9095674

[16] Tefferi A (2014). The prognostic advantage of calreticulin mutations in myelofibrosis might be confined to type 1 or type 1-like CALR variants. Blood.

[17] Lasho TL, Finke CM, Tischer A, Pardanani A, Tefferi A (2018). Mayo CALR mutation type classification guide using alpha helix propensity. American journal of hematology.

[18] Forbes SA (2017). COSMIC: somatic cancer genetics at high-resolution. Nucleic acids research.

[19] Stenson PD (2017). The Human Gene Mutation Database: towards a comprehensive repository of inherited mutation data for medical research, genetic diagnosis and next-generation sequencing studies. Human genetics.

[20] Kralovics R (2005). A gain-of-function mutation of JAK2 in myeloproliferative disorders. N Engl J Med.

[21] Pikman Y (2006). MPLW515L is a novel somatic activating mutation in myelofibrosis with myeloid metaplasia. PLoS medicine.

[22] Tefferi A (2014). Type 1 versus Type 2 calreticulin mutations in essential thrombocythemia: a collaborative study of 1027 patients. American journal of hematology.

[23] Kim SY (2015). CALR, JAK2, and MPL mutation profiles in patients with four different subtypes of myeloproliferative neoplasms: primary myelofibrosis, essential thrombocythemia, polycythemia vera, and myeloproliferative neoplasm, unclassifiable. American journal of clinical pathology.

[24] Rabade N (2018). Molecular genetics of BCR-ABL1 negative myeloproliferative neoplasms in India. Indian journal of pathology & microbiology.

[25] Ojeda MJ (2018). CALR, JAK2 and MPL mutation status in Argentinean patients with BCR-ABL1- negative myeloproliferative neoplasms. Hematology (Amsterdam, Netherlands).

[26] Wang J (2017). JAK2, MPL, and CALR mutations in Chinese Han patients with essential thrombocythemia. Hematology (Amsterdam, Netherlands).

[27] Geyer JT, Orazi A (2016). Myeloproliferative neoplasms (BCR-ABL1 negative) and myelodysplastic/myeloproliferative neoplasms: current diagnostic principles and upcoming updates. International journal of laboratory hematology.

[28] Tefferi A, Barbui T (2017). Polycythemia vera and essential thrombocythemia: 2017 update on diagnosis, risk-stratification, and management. American journal of hematology.

[29] Zamora L (2015). Calreticulin mutations are not present in patients with myeloproliferative chronic myelomonocytic leukemia. Annals of hematology.

[30] Hu Z (2019). Utility of JAK2 V617F allelic burden in distinguishing chronic myelomonocytic Leukemia from Primary myelofibrosis with monocytosis. Human pathology.

[31] Guglielmelli P (2015). Validation of the differential prognostic impact of type 1/type 1-like versus type 2/type 2-like CALR mutations in myelofibrosis. Blood cancer journal.

[32] Rumi E (2014). JAK2 or CALR mutation status defines subtypes of essential thrombocythemia with substantially different clinical course and outcomes. Blood.

[33] Lasho TL, Elliott MA, Pardanani A, Tefferi A (2014). CALR mutation studies in chronic neutrophilic leukemia. American journal of hematology.

[34] Smaili W (2017). CALR gene mutational profile in myeloproliferative neoplasms with non-mutated JAK2 in Moroccan patients: A case series and germline in-frame deletion. Current research in translational medicine.

[35] Lin Y (2015). The Prevalence of JAK2, MPL, and CALR Mutations in Chinese Patients With BCR-ABL1-Negative Myeloproliferative Neoplasms. American journal of clinical pathology.

[36] Forbes SA (2016). COSMIC: High-Resolution Cancer Genetics Using the Catalogue of Somatic Mutations in Cancer. Current protocols in human genetics.

[37] Barbui T, Finazzi G, Falanga A (2013). Myeloproliferative neoplasms and thrombosis. Blood.

[38] Tefferi A, Barbui T (2019). Polycythemia vera and essential thrombocythemia: 2019 update on diagnosis, risk-stratification and management. American journal of hematology.

[39] McMahon B, Stein BL (2013). Thrombotic and bleeding complications in classical myeloproliferative neoplasms. Seminars in thrombosis and hemostasis.

[40] Kaifie A (2016). Bleeding, thrombosis, and anticoagulation in myeloproliferative neoplasms (MPN): analysis from the German SAL-MPN-registry. Journal of hematology & oncology.

[41] Tefferi A, Elliott M (2007). Thrombosis in myeloproliferative disorders: prevalence, prognostic factors, and the role of leukocytes and JAK2V617F. Seminars in thrombosis and hemostasis.

[42] Abdulkarim K, Samuelsson J, Johansson P, Andreasson B (2017). Risk factors for vascular complications and treatment patterns at diagnosis of 2389 PV and ET patients: Real-world data from the Swedish MPN Registry. European journal of haematology.

[43] Kc D, Falchi L, Verstovsek S (2017). The underappreciated risk of thrombosis and bleeding in patients with myelofibrosis: a review. Annals of hematology.

[44] Barbui T (2010). Thrombosis in primary myelofibrosis: incidence and risk factors. Blood.

[45] Cervantes F (2006). Frequency and risk factors for thrombosis in idiopathic myelofibrosis: analysis in a series of 155 patients from a single institution. Leukemia.

[46] Barosi G (2012). Evidence that prefibrotic myelofibrosis is aligned along a clinical and biological continuum featuring primary myelofibrosis. PloS one.

[47] Rotunno G (2014). Impact of calreticulin mutations on clinical and hematological phenotype and outcome in essential thrombocythemia. Blood.

[48] Langabeer SE (2016). Chasing down the triple-negative myeloproliferative neoplasms: Implications for molecular diagnostics. Jak-stat.

[49] Grinfeld J (2018). Classification and Personalized Prognosis in Myeloproliferative Neoplasms. N Engl J Med.

[50] Cimen Bozkus C (2019). Immune Checkpoint Blockade Enhances Shared Neoantigen-Induced T-cell Immunity Directed against Mutated Calreticulin in Myeloproliferative Neoplasms. Cancer discovery.

[51] Holmstrom MO (2018). The calreticulin (CALR) exon 9 mutations are promising targets for cancer immune therapy. Leukemia.

[52] Holmstrom MO, Riley CH, Svane IM, Hasselbalch HC, Andersen MH (2016). The CALR exon 9 mutations are shared neoantigens in patients with CALR mutant chronic myeloproliferative neoplasms. Leukemia.

[53] Klausen U (2018). Novel Strategies for Peptide-Based Vaccines in Hematological Malignancies. Frontiers in immunology.

[54] Stauffer Larsen T (2013). Long term molecular responses in a cohort of Danish patients with essential thrombocythemia, polycythemia vera and myelofibrosis treated with recombinant interferon alpha. Leukemia research.

[55] Zak KM (2017). Structural Biology of the Immune Checkpoint Receptor PD-1 and Its Ligands PD-L1/PD-L2. Structure (London, England: 1993).

