# Supplementary material for: CALR mutations in a cohort of JAK2 V617F negative patients with suspected myeloproliferative neoplasms
Source: Sci Rep. 2019 Dec 27;9:19838. doi: 10.1038/s41598-019-56236-x (PMC6934448; doi:10.1038/s41598-019-56236-x)
Supplement: Supplementary file 1 — Supplementary Information [file 41598_2019_56236_MOESM1_ESM.pdf]

CALR mutations in a cohort of *JAK2* V617F negative patients with suspected myeloproliferative neoplasms

Authors: Tanja Belcic Mikic[1,2,*], Tadej Pajic [1,3], Matjaz Sever [1,2]

1 Department of Haematology, University Medical Centre Ljubljana, Zaloska 7, 1000 Ljubljana, Slovenia

2 Faculty of Medicine, University of Ljubljana, Korytkova 2, 1000 Ljubljana, Slovenia

3 Faculty of Medicine, University of Maribor, Taborska ulica 8, 2000 Maribor, Slovenia

* ORCID ID 0000-0002-0092-5682

Correspondence:

Tanja Belcic Mikic, MD

Department of Haematology, University Medical Centre Ljubljana, Zaloska 7, 1000 Ljubljana, Slovenia

Faculty of Medicine, University of Ljubljana, Korytkova 2, 1000 Ljubljana, Slovenia

email: tbelcic@gmail.com

telephone number: +38640500289

Supplementary Table S1: The list of diagnosis in myeloproliferative neoplasm (MPN) suspected patients

Diagnosis	*No. of patients*
Secondary/reactive erythrocytosis/leucocytosis/thrombocytosis	380
ET (by WHO** criteria 2008)*	10
*Primary myelofibrosis (by WHO** criteria 2008)*	4
*Suspected clonal ET**	32
Myelodysplastic syndrome	8
Mastocytosis	3
Haemochromatosis	2
Anaemia (Chronic inflammatory, iron deficiency, aplastic, thalassemia)	15
Splenomegaly - unclassifiable	5
Hypersplenism	3
Chronic lymphocytic leukaemia	3
Haemophilia	2
Eosinophilia	6
*MPN-unclassifiable (by WHO** criteria 2008)*	10
*Polycythaemia vera (by WHO** criteria 2008)*	1
Suspected primary polycythaemia	10
Lymphoma	7
Acute myeloid leukaemia	4
Chronic myelomonocytic leukaemia	2
Thrombocytopenia - undetermined	9
Portal vein thrombosis	1
*Chronic neutrophilic leukemia (by WHO** criteria 2008)*	1
MDS/MPN--unclassifiable	2
Other #	4

*essential thrombocythemia, **World Health Organisation, # secondary myelofibrosis, monoclonal gammopathy of undetermined significance, leukopenia due to toxic effect of drugs, diagnosis not found.
